# Ocular Morbidity—A Critical Analysis to Improve Outpatient Services in an Eye Department in a Sub-Saharan Megacity

**DOI:** 10.3390/jcm10173791

**Published:** 2021-08-25

**Authors:** Edith Mukwanseke, Janvier Kilangalanga, Flavien Lutete, Adrian Hopkins, Rudolf F. Guthoff, Stefanie Frech

**Affiliations:** 1 Centre de Formation Ophtalmologique Pour l’Afrique Centrale, Eye Department, Saint Joseph’s Hospital, Kinshasa P.O. Box 322, Democratic Republic of the Congo; edithodio@yahoo.fr (E.M.); kilangalanga@yahoo.fr (J.K.); lutetelongo@gmail.com (F.L.); 2 National Programme for Eye Health and Vision, Kinshasa P.O. Box 322, Democratic Republic of the Congo; adrianhopkinsconsulting@gmail.com; 3 Department of Ophthalmology, Rostock University Medical Centre, 18057 Rostock, Germany; rudolf.guthoff@med.uni-rostock.de

**Keywords:** ocular morbidity, eye care management, Kinshasa

## Abstract

The aim of this study was to analyse outpatient services in an ophthalmic clinic of a church-run hospital providing secondary level care in an African megacity, paying special attention to the poorest users of the services. The range of examination was reviewed from 500 patient records of all ages consecutively chosen on random days attending the outpatient department for the first time in order to optimize workflow and to analyse the offered treatment modalities. Mean age was 41.9 ± 21.9 years, and 53.6% of the patients were female. Of the patients, 74.8% presented with visual impairment. The most frequent findings were refractive errors (35.8%), presbyopia (21.2%), allergic conjunctivitis (14.0%), cataract (13.2%) and glaucoma (6.4%). Patient management consisted of optical treatment (49.6%), surgery (11.4%) and medical treatment (39.0%). These results show the importance of the demand in refractive services and the need to train specific service providers. Knowing the frequencies of common conditions enables more appropriate diagnostic and treatment strategies, e.g., the importance of refractive errors, and should lead to improvements in training, staffing, therapeutics and patient outcomes. This approach can be applied to many other outpatient services and should be evaluated in light of the city’s impoverished health outreach and educational situation.

## 1. Introduction

The epidemiological profile of ocular pathologies varies in different regions of the world and is influenced by diverse factors like ethnic, geographical, climatic, socioeconomic and cultural factors [1]. The term ocular morbidity describes conditions creating both visual impairment and nonvisual pathology [2].

According to the World Health Organization (WHO) and the recent data of Blindness and Vision Impairment Collaborators, in 2020, 295 million people globally were affected by moderate and severe visual impairment, and 43.3 million were blind. Major causes of visual impairment are uncorrected refractive errors and cataract, while the main cause of blindness is cataract (45.5%) followed by glaucoma [3,4]. Ninety percent of visually impaired people live in low and middle-income countries [5]. The greatest gap between need and available eye services has been identified in sub-Saharan Africa [6]. Reasons for the high numbers of visually impaired people are manifold and include poverty and a lack of access to eye services [7]. Factors for the high burden of visual impairment are weighted differently depending on the development status of the population and include patient-related factors, health system-related factors and infrastructure-related factors [8]. The decrease of the total number of individuals with visual impairment from 26.8 million in 2002 to 26.3 million in 2010 in Africa may be attributed to the achievements of VISION 2020.

Nonvisual ocular morbidities were not always the primary focus, as the driving force of investigations is principally on the reduction of visual impairment and blindness. Although they do not necessarily lead to blindness, these morbidities cause distress and result in demand for health services. Ocular morbidities may also result in a decreased ability to perform activities of daily life, and should be investigated accordingly [5,9].

The DRC is a vast country with approximately 90 million inhabitants depending on different estimations. Gross Domestic Product (GDP) per capita in 2018 was 562 USD. DRC is a low-income country, with 8 of 10 inhabitants living for under one dollar per day. The Health System is based on three tiers, national, provincial and the health districts. In general, health care is based on a four-level pyramid, with the first level being community health centres providing basic care by nurses. The second level comprises reference health centres or district hospitals with general physicians, and the third—provincial hospitals offering special care. University hospitals comprise the fourth level [10]. The government increased health care spending to almost nine percent of the overall budget in 2015 compared to three-point-four percent in 2011. Still, government spending on health care continues to be among the lowest in the world [11]. The result is poor access to health care and a high unmet need for health care in large parts of the country. Some decent health care is provided in facilities that get support from governmental or nongovernmental organizations [12]. There is a National Plan for Eye Care and Vision, but it is underfunded and with limited human resources. Some provinces still do not have an eye service. Kinshasa, the capital, is a city with an estimated population of between 8 and 10 million. Most ophthalmologists are present in the capital but principally they run small private clinics and do not do surgery. There is a University Clinic, but it is inaccessible for much of the population, and most eye care activities are done at two charity hospitals. The knowledge about the prevalence of ocular morbidities of Saint Joseph’s Hospital could influence the planning and management of eye care services.

The purpose of this study was to determine the epidemiological profile of ophthalmic pathology in an outpatient department of an African megacity, namely the outpatient department at Saint Joseph’s Hospital, Kinshasa, Democratic Republic of Congo (DRC), which may be applicable in other low- and middle-income countries, where there is limited prevalence data. Although a more detailed study is now needed, the results have shown the importance of proper management of refractive errors in this particular location, which have not been a priority to date.

## 2. Materials and Methods

### 2.1. Settings and Catchment Area

Our study is a descriptive study, carried out at Saint Joseph’s Hospital, Kinshasa, DRC. The hospital is a charity district hospital owned by the Catholic Diocese located in the Limete district of the city, with patients mainly from low-income families. It is integrated into the health system and is one of the district hospitals of the city. Patients have to contribute to the costs, but they are often lower than those at government hospitals. Situated about 10 km away from the city centre where private hospitals take care of high-income patients, the ophthalmology department has a bed capacity of 22. Yearly consultations are around 31,200 with 1200 cataract surgeries and an overall number of 3100 surgical cases.

During the last 20 years, two ophthalmological subspecialties have been developed in cooperation with the Rostock University Eye Hospital, Germany [13,14]. These are paediatric ophthalmology with special emphasis on childhood cataract blindness and a centre for diabetic retinopathy and laser treatment as a part of extensive diabetic care units coordinated by the catholic medical network (Catholic Diocesan Office for Medical Work, BDOM). Beside these two units, there is a general ophthalmology outpatient clinic. Inclusion criteria also included referral from other medical services from the hospital or from outside the hospital. Exclusion criteria included former patients who came back to the clinic for follow up visits and hospitalized patients.

### 2.2. Measured Variables

For this study, the patient records of 500 patients of all ages consecutively chosen on random days attending the outpatient department for the first time between September and November 2019 were reviewed. A data collection sheet, specifically designed for the study with questions concerning demographic data, main disorders of the patients, diagnoses and decisions, was used to assemble the data from a review of the patient charts.

Clinical examination included visual acuity measured with the Snellen chart and the E test for illiterate people. For children, the LEA test symbols were used. The anterior segment was examined using the slit lamp; the fundus was assessed with the direct ophthalmoscope or a 90 dioptre lens in conjunction with a slit lamp for stereoscopic view. In questionable cases, pupil dilatation was used to get a clearer fundus view where necessary. Intraocular pressure (IOP) was measured with the indentation tonometer by Schiotz or applanation tonometry by Goldman tonometer or Perkins. Children were examined with an Icare tonometer to measure IOP. Depending on the case, additional examinations were performed such as automatic refraction, B scan ultrasound, biometry and optical coherence tomography. Cataract was defined by lens opacification causing a reduction of visual acuity to 6/60 or less. Glaucoma was defined by an IOP of 25 mmHg or more and/or typical changes of the optic disc. The assessment of optic disc morphology is important for the diagnosis. For example, Africans have a larger optic disc than Europeans as well as a deeper mean cup depth, which must be taken into account in the diagnosis of glaucoma [15].

### 2.3. Statistical Analysis

All data were stored and analysed using the SPSS statistical package 20.0 (SPSS Inc. Chicago, IL, USA). Descriptive statistics were computed for continuous and categorical variables. The statistics computed included mean and standard deviations (SD) of continuous variables and are presented as mean ± SD, frequencies and percentages of categorical factors.

The variables analysed were age, sex, complaints at presentation, symptoms, diagnoses, specialized examinations, laboratory examinations and proposed treatment. Confidence intervals of 95% (95%-CI) for important parameters are reported to show the reliability of the point estimates. A major factor determining the length of a confidence interval is the size of the used sample. The worst case would be a point estimate of 50%. In this case, we wanted to achieve an interval length of about 8–10 percent. It was possible with 500 people taking part in the survey. The sample size was determined by using the study planning software “nQuery Advisor^®^ 7.0” (nQuery (2017)) (Statistical solutions, Saugus, USA).

## 3. Results

**Figure 1:**
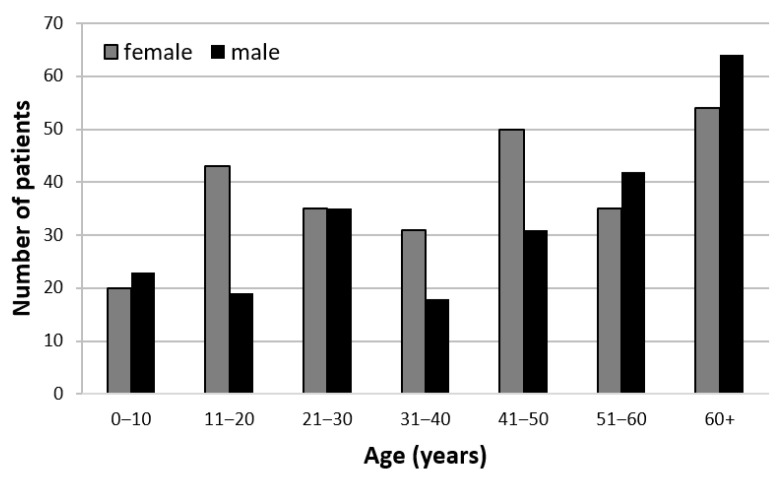
Age and sex distribution of the study sample.

Out of a total of 500 examined patients, 268 were female (53.6%). The mean age was 41.9 years (ranging from 1 to 95 years). Figure 1 presents the age and sex distribution of the study sample.

### 3.1. Symptoms

Visual impairment was the main symptom (74.8%; 95%-CI: (71.0; 78.6)), followed by pain (27.8%; 95%-CI: (23.9; 31.7)) and epiphora (25.8%; 95%-CI: (22.0; 29.6)) (Table 1). For various reasons, 12.6% of the patients were transferred from other health facilities in the town for treatment.

### 3.2. Diagnoses

Distance refractive errors were the first cause of ocular morbidity with 24.4% myopia (95%-CI: (20.6; 28.2)) and 11.4% hyperopia (95%-CI: (8.6; 14.2)), followed by 21.2% presbyopia (95%-CI: (17.6; 24.8)), allergic conjunctivitis (14.0%; 95%-CI: (11.0; 17.0)), cataract (13.2%; 95%-CI: (10.2; 16.2)), glaucoma (6.4%; 95%-CI: (4.3; 8.5)) and other pathologies of the conjunctiva (pterygium, pinguecula, nevus and others). Pathologies of the posterior segment consisted of diabetic retinopathy (1.2%), hypertensive retinopathy (1.0%), chorioretinitis (0.8%), retinal venous abnormality (0.8%), retinal detachment (0.6%), degeneration of the vitreous (0.4%), vitritis (0.2%) and vitreous haemorrhage (0.2%). Table 2 shows the frequencies of the ocular diagnoses. As some patients had more than one diagnosis, the total number of diagnoses is higher than the total number of patients participating in the study.

**Figure 2:**
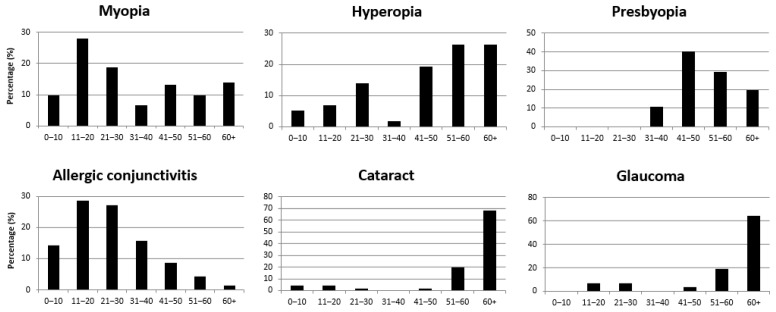
Percentage distribution of the most common diagnosed eye diseases by age groups.

Out of the six most frequent ocular diagnoses, the distribution within the different age groups of presented patients was calculated (Figure 2). Myopia was most common in the age groups of 11–20 years, (27.9%), 21–30 years (18.9%) and 60+ years (13.9%), but myopic patients were found within all age groups. Myopic persons with more than −1.50 dioptres were included. Hyperopia increased with age, with the highest rates for the ages 41–50 (19.3%), 51–60 (26.3%) and 60+ (26.3%). Presbyopia was mainly diagnosed in patients aged 41–50 (40.2%), with decreasing percentages for the age groups of 51–60 (29.4%) and 60+ (19.6%). Allergic conjunctivitis was highest in the age groups of 11–20 (28.6%) and 21–30 (27.1%) years and decreased further in older patients. Cataract was mainly diagnosed in the age group of 51–60 (19.7%) and 60+ (68.2%), whereas glaucoma was mostly diagnosed over the age of 60+ (64.5%).

### 3.3. Additional Examinations

Saint Joseph’s Hospital Ophthalmology Department is well-equipped for most routine diagnostic procedures. In about half of the patients, the initial ophthalmological examination was followed by more specific diagnostic procedures which nearly always involved extra costs. Automated refractometry made up almost 30% of the examinations, followed by B scan ultrasound with 10.6%, optical coherence tomography (OCT) (2.6%) and A scan ultrasound (2.0%) (Table 3).

### 3.4. Treatments

Table 4 shows the therapeutic recommendations offered by Saint Joseph’s Hospital in broad groups. The main option was optical correction of patients (49.6%), followed by medical (39.0%) and surgical (11.4%) treatment. Optical correction included the prescription of glasses and was partly delivered by an in-house optical workshop. Surgical options are available at the hospital and are mainly in the field of cataract and glaucoma (small incision cataract surgery with lens implantation (SICS), trabeculectomy with mitomycin application.

Table 5 puts our results in the context of other studies dealing with ocular morbidity in different parts of the world and provides a comparison of these with the literature implemented in this manuscript.

## 4. Discussion

Our study showed a small difference in the presentation of male (46.4%) and female (53.6%) patients to the outpatient department at Saint Joseph’s Hospital. Similar numbers in gender were also noted in ocular morbidity studies conducted in India [21,23] with 46.9% and 46.5% males, Ethiopia [16] with 49.5% males, Nigeria [25] with 51.3% males and in the study of Thomson and Chumbley [26] in 1984 where 55.4% of patients were women and 44.6% were men. In the study of Oladigbolu et al. [27], almost 20.0% more males presented with eye diseases. In the study of Rizyal et al. [17], 67.1% of the patients were female and 32.9% were male. These inconsistent results imply that the gender gap is not very pronounced when looking at patient pools of all ages. Having a closer look at the individual age groups, however, the result shows that in the age groups of 11–20 years and 41–50 years, the number of female patients was considerably higher than that of male patients (Figure 1). It has been suggested that girls in the younger age group are more concerned about aesthetic factors, such as red eyes in conjunctivitis, which is a major diagnosis in that age group. For patients aged 41–50, men often find less time and have less need for near vision aids in everyday working life, dominated by outdoor activities. Women are more concerned about details in daily home-life and making and repairing clothing. For patients aged 41–50, similar results were found by Rizyal et al. [17], with 16.6% versus 10.8%.

Saint Joseph’s Hospital Eye Department plays a double role in the ophthalmological field of Kinshasa with two subspecialties (as mentioned in Methods) as well as a general eye outpatient department serving as a comprehensive eye care centre for a population of approximately two million, which is demonstrated by the broad spectrum of diagnoses. In societies with developed health care systems, these patients most likely will be seen and taken care of by numerous ophthalmologists and optometrists practicing in decentralized units. In general, it was observed that diagnoses affecting the lens increased with age and diagnoses affecting the conjunctiva decreased with age, which has also been described previously [9].

In this study, the prevalence of refractive errors was 35.8%. This number was approximately one-third higher compared to those from the Nepal study of Rizyal et al. [17] with 22.5%, the Indian study of Singh A et al. [19] with 21.6% and the Nigeria study of Ukponmwan [1] with 23.1%. Similar numbers were reported in the Indian study of Baldev et al. [21] with 32.6%. Singh et al. [23] reported an even higher rate of refractive errors with 40.8%, but as the study was performed in patients aged 50+ years, these numbers are self-explanatory. When looking at specific age groups, the highest number of refractive errors in this study with 34.9% was at the age of 11–20, decreasing with older patients. As expected, myopia, which starts in childhood and progresses with age [28], was predominantly diagnosed in this younger age group (56.6% below the age of 30), whereas hyperopia was symptomatic at all ages, with its peak at age 50 and older. Similar to our study with 34.9% of refractive errors at young ages, a study investigating children in Osogbo, Nigeria by Isawumi et al. [24] analysed a very similar number of refractive errors with 27.8% in children aged 3–16.

Presbyopia was most frequently diagnosed at age 41–50 with 40.2% and decreased again with older age. Overall, presbyopia was diagnosed in 21.2%, similar to 25.1% detected by Kimani et al. [9] in Uganda. In 2015, the global unmet need for presbyopia correction was estimated to be 45.0%. People are more likely to have an adequate optical correction if they live in an urban area of a more developed country. These data correspond with our results for patients aged 41–50 as well as with the results from the Uganda study of Kamali et al. [22], who describe the highest amount of presbyopia with 48.0%.

In total, 23.2% conjunctiva-associated pathologies were analysed. As the time of data acquisition was from September to November (end of the dry season with the start of the rainy season in Kinshasa), allergic conjunctivitis dominated, as a high load of pollen was usual and may have led to some selection bias, although heat and dust are contributing factors. It accounted for 14.0% of diagnoses with a peak of 28.6% at age 11–30, decreasing with older ages. Nevertheless, higher numbers were shown for other areas of sub-Saharan Africa like Nigeria (19.9%) and Uganda (20.0%) [1,22]. Unspecified conjunctivitis was described by Zalelem et al. [16] with 29.0%, by Khan et al. [20] with 26.0% and by Kimani et al. [9] with 31.0%. Concerning infectious conjunctivitis, Kamali et al. [22] found 8.0% in Uganda, compared to 3.2% in our study. Other conjunctival pathologies were described by Rizyal et al. [17] in Nepal with 10.8%, dominated by pingueculae and pterygium, which was almost double the number of our results with 6.0%.

Cataract was mostly seen in higher ages (50 and older), with a total number of 13.2% for all ages. Three more studies performed in countries of sub-Saharan Africa revealed similar numbers. Those are the Ethiopia study by Zalelem et al. [16] with 16.3%, the Nigeria study of Ukponmwan [1] with 15.9% and the Uganda study of Kamali et al. [22] with 9.0%. Numbers lower than 5% were illustrated by Khan et al. [20] with 4.3% and by Khadse et al. [18] with 4.8%. In contrast, numbers higher than 40% were reported by three Indian studies with 40.4%, 43.7% and 41.9% [19,21,23].

In the black African population, glaucoma prevalence (mostly chronic open-angle glaucoma) is higher than that in Caucasian populations. It starts at younger ages, and the progression is more pronounced. These factors and the limited diagnostic and therapeutic possibilities, especially in rural areas of Africa, are the reasons why glaucoma-related blindness is more common in Central Africa than in other parts of the world [29,30,31,32].

Glaucoma was diagnosed in 6.4% of patients, mainly in patients aged 50 and older. In a Northern Nigeria study of Oladigbolu et al. [27], the prevalence was found to be 1.9%, which was less than what was found in the African population of other regions like Cameroon (8.2%) [33] and Ghana (8.5%) [34] or elsewhere in Nigeria (11.9%) [1]. However, it can be explained by the fact that the majority of patients in the study were younger than 40 years. The Nigeria Blindness Survey of Kyari et al. [35] referred to 5.0% of survey participants as being affected by glaucoma, which is about the same percentage found in this study, and the authors described strategies to improve glaucoma management. Similar numbers were also described by Baldev et al. [21] with 3.7% and Singh et al. [19] with 4.8%.

According to recommended treatments, optical treatment represented 49.6%, followed by medical treatment at 39.0%. Referrals for special examinations in this study were dominated by refractometry (29.4%), followed by B scan ultrasound (10.6%). This study incorporated patients from all age groups, which is the reason for the higher percentage of refractive errors (35.8%). In our study, refractive errors include myopia (24.4%) and hyperopia (11.4%), but not presbyopia (21.2%). This fact might be different to the results published by others; therefore, it is difficult to directly compare prevalences. As demonstrated in Table 3, it is obvious that 29.4% of the study cohort have been sent to automated refractometry (147 patients). The extensive use and the costs for its use suggests that better trained ophthalmology staff, such as Techniciens Supérieur en Ophtalmologie (TSO) and optometrists could improve the service at lower cost by being better-trained in manual refraction. Prescription of glasses should be provided and be possible without refractometry. Reasons to perform automated refractometry should be clearly specified by the requester.

Following the critical evaluation of Salman et al. [36] in 2006, ocular ultrasound is only required when the fundus is not accessible and special risk factors like posterior synechia and signs of panuveitis are present. As ultrasound is rarely available and is related with extra costs for the patients. A preselection of patients based on diagnoses before performing an ultrasound could be a more efficient way to organize ocular examinations. The low number of patients with diabetic retinopathy and paediatric cataract could be explained by the fact that there are subspecialized units for diabetes-related eye pathology and paediatric ophthalmology in the same hospital next to the general outpatient clinic. Toxoplasmic serology was requested in 1.2% of cases. These last three variables are not mentioned in other studies. Well-trained optometrists could handle a large proportion of the workload in outpatients by setting up optometry examination areas, as at the moment, all ophthalmic nurses and even ophthalmologists spend their valuable time dealing with refractive errors. Doctors do not necessarily need to do the examination and they would be available for more complex medical and surgical tasks. This task shifting might be especially considered, as the number of health care workers in the DRC is approximately 0.09 physicians to 1000 individuals, much less than in many other countries, such as the United States with almost 3 physicians to 1000 individuals or more than 4 physicians to 1000 individuals in Italy [11]. There would still need to be a general examination including glaucoma control, but with the increasing prevalence of myopia, developing optometry and establishing a priority treatment strategy could become more important. In addition, the reduction of the demand for follow-up that can be effectively treated at the first presentation with a tailored eye care service to provide a cost-effective treatment should also be considered. For the training of nonsurgical skills, there are ophthalmologists and optometrists in industrialised countries prepared and willing to teach on-site and to take over twinning partnerships. Offering the abovementioned customized subspecialties, training programs for TSOs and ophthalmologists and improvement of access to service and the definition of interfaces between the work of the health care personnel requires working hand-in-hand and improving the quality of service.

## 5. Conclusions

In conclusion, the study provides information about the frequency of eye pathologies of patients presenting at a clinic in the periphery of a sub-Saharan megacity. Refractive errors were the most common, followed by presbyopia (21.2%), allergic conjunctivitis (14.0%) and cataracts (13.2%). It is clear, particularly in this urban environment, that refractive errors contribute significantly to visual impairment and that the leading causes of ocular morbidity were preventable and/or treatable. A future study investigating epidemiological factors and therefore giving deeper knowledge of the given circumstances would be useful to further improve patient care. The introduced data collection sheet could now prove beneficial for this purpose.

As a result of this work, recommendations can be made both for the eye clinic as well as more generally for other departments in order to improve the patients’ experience and to build confidence:

1. The data can be used to optimise outpatient management in Saint Joseph’s Hospital. The department needs to increase its capacity to manage people with refractive errors more efficiently. Concerning refraction measurement, retraining to perform a quick and precise refraction that is not dependent on high-tech instruments is advisable and would save patients money and time. Establishing a refraction unit (optical workshop) where optometrists would take care of half of the patients every day to let doctors deal with core complex eye diseases to run services more efficiently, could be a practical solution. These results may also be applicable to other clinics in an environment with limited resources, both economic (gross national income (GNI) less than $1025 per capita [37]) and human (less than two ophthalmologists per million inhabitants).
2. The most relevant pathologies (refractive errors, glaucoma) need to be taken into account. As pathologies differ with age, the affected population should also be taken into account when planning eye care services.
3. To improve and to manage health care service more effectively, different strategies were discussed by others, like integrating social workers into care systems [2], forming a framework which stakeholders will utilize to train personnel to prevent blindness [27] or restructuring and shifting parts of care away from expensive services to primary care levels [9].
4. Maketa and colleagues [38] also showed that the population is willing to use public health services if they are functional with fair, affordable and predictable costs. Presently, one of the major obstacles is a lack of confidence towards all kinds of medical care. This is partly due to the low level of education of the population, leading to the fact that even costless medical help and support is omitted. A key factor in such a process is to verify that the proposed intervention addresses a health issue that is acknowledged and considered relevant by the community [21]. It is therefore important to improve overall understanding of community and patient demands regarding health-related interventions.
5. In addition to improving health care to reduce health inequities, government and international actors must ensure that communities are truly informed about health programs, their rationale and their risks/benefits, as mentioned by Maketa et al. [38]. To improve acceptance and general access to health services, direct involvement of community members seems to be the best way.
6. Our findings suggest that there is an unmet need for glasses, as three of the five most relevant findings required spectacle correction. About 50% of all patients are treated with a prescription for glasses. It might be worth considering whether, e.g., prefabricated presbyopic spectacles, which cost less than two US dollars, should be supplied directly with or without extra charges.
7. No patient should leave the clinic without a clear management strategy, whether it be spectacles or suggestions for medical, laser or surgical treatment, ophthalmological or general. In accordance with Stasse et al. [12], our experience demonstrates that it is possible to improve health district regulation by conditioning the financial support to a more rational use of available resources. Glaucoma is the second leading cause of blindness, and the prevalence is higher in Africa than in other regions in the world. Even IOP measurement and evaluation of the optic disc to better diagnose glaucoma could be performed after intensive training and regular retraining courses by TSOs, the equivalent of Ophthalmic Medical Assistants in other countries or optometrists, given the fact that there are well-trained and qualified ophthalmologists in place for further advice if required.

## Figures and Tables

**Table 1:** Items most frequently noted in the analysed medical records.

	No	%
Visual impairment	374	74.8
Pain	139	27.8
Epiphora	129	25.8
Red eye	67	13.4
Corneal opacities	28	5.6
Trauma	9	1.8

**Table 2:** Frequencies of ocular diagnoses.

Diagnoses	No	%
Myopia	122	24.4
Presbyopia	106	21.2
Allergic conjunctivitis	70	14.0
Cataract	66	13.2
Hyperopia	57	11.4
Primary open-angle/juvenile glaucoma	29	6.4
Other conjunctival pathologies	30	6.0
Infectious conjunctivitis	16	3.2
Other lid pathologies	12	2.4
Keratitis (ulcerative and not ulcerative)	14	2.3
Other corneal pathologies	8	1.6
Ocular contusion	7	1.4
Iridocyclitis	7	1.4
Diabetic retinopathy	6	1.2
Hypertensive retinopathy	5	1.0
Perforative ocular globe trauma	5	1.0
Chorioretinitis	4	0.8
Retinal venous abnormalities	4	0.8
Retinal detachment	3	0.6
Entropion	2	0.4
Lid inflammation and infection	2	0.4
Vitreous degeneration	2	0.4
Corneal opacification	1	0.2
Ectropion	1	0.2
Ptosis	1	0.2
Other lens pathologies	1	0.2
Vitritis	1	0.2
Vitreous haemorrhages	1	0.2

**Table 3:** Specialized examinations.

Ordered Addition Examination	No	%
Automatic refractometry *	147	29.4
B scan ultrasound **	53	10.6
Optical coherence tomography (OCT) ***	13	2.6
A scan ultrasound	10	2
Visual field	8	1.6
Angiography	4	0.8
Retinal photography	3	0.6
Laboratory tests		
Toxoplasmosis serology	6	1.2
VIH serology	2	0.4

Main indications: * poor patient cooperation during visual acuity check or retinoscopy (27), reduction of vision not explained by optical media and ophthalmological finding (120), ** opacification of cornea (4), lens (30), vitreous (19), *** ill-defined macular lesion (10), suspicion of posterior pole retinal detachment (3).

**Table 4:** Treatment recommendations.

Treatments	No	%
Optical	248	49.6
Medical	195	39.0
Surgical	57	11.4
No treatment	2	0.4
Laser	1	0.2
Transfer	2	0.4

**Table 5:** Comparison of various studies on ocular morbidity including different regions and ages of patients. Numbers are given in percentage. ^†^ Conjunctival degeneration (pterygium, pinguecula), ^‡^ inclusive presbyopia, OM: ocular morbidity.

Study	Mukwan-seke, 2020 Kinshasa DRC	Mehari 2013 [16] Ethopia	Ukpon-mwan 2013 [1] Nigeria	Rizyal 2010 [17] Nepal	Khadse 2014 [18] India	Singh 2012 [19] India	Khan 2015 [20] Saudi Arabia	Kimani 2013 [9] Kenya	Baldev 2017 [21] India	Kamali 1999 [22] Uganda	Singh 1997 [23] India	Isawumi 2016 [24] Nigeria
Patients	500	214	7220	395	525 (212 OM)	9736 (933 OM)	1110	3691 (563 OM)	450	173	903	180
Age	all ages	all ages	all ages	all ages	all ages	all ages	all ages	all ages	60+	13–65+	50	3–16
Male	46.4	50.5	49.6	32.9	51.4	46.4		43.1	46.4	48.0	53.1	33.9
Female	53.6	49.5	50.4	67.1	48.6	53.6		56.9	53.6	52.0	46.9	66.1
Myopia	24.4	3.3			5.1							21.7
Presbyopia	21.2	15.4			19.8			25.1		48.0		
Allergic conjunctivitis	14.0	12.1	19.9							20.0		
Cataract	13.2	16.3	15.9	17.5	4.8	41.9	4.3		43.7	9.0	40.4	
Hyperopia	11.4	2.3			2.7							6.1
Glaucoma	6.4	3.3	11.9		0.2	4.8	2.3		3.7		3.1	
Other conjunctival pathologies	6.0			10.8 ^†^								
Infectious conjunctivitis	3.2									8.0		
Refractive errors	35.8	7.9	23.1	22.5		21.6	27.7 ^‡^		32.6		40.8	27.8

## Data Availability

The datasets used and/or analysed during the current study are available from the corresponding author upon reasonable request.
